# From Structure to Function of Promoters and 5′UTRs in Maize

**DOI:** 10.3390/ijms27010548

**Published:** 2026-01-05

**Authors:** Nikita V. Sytov, Vladimir V. Choob, Sileshi Nemomissa, Alexander S. Mishin, Maxim M. Perfilov

**Affiliations:** 1 Shemyakin-Ovchinnikov Institute of Bioorganic Chemistry, Russian Academy of Sciences, Moscow 117997, Russia; nvsytov@gmail.com (N.V.S.); max@planta.bio (M.M.P.); 2 Botanical Garden of Lomonosov Moscow State University, Vorobievy Gory 1 b.12, Moscow 119234, Russia; 3 Department of Plant Biology and Biodiversity Management, Addis Ababa University, Addis Ababa P.O. Box 3434, Ethiopia

**Keywords:** maize, gene expression, promoter, 5′ untranslated region, biotechnology

## Abstract

As a cornerstone of global agriculture, maize (*Zea mays*) is a crucial component of sustainable food systems and industrial uses. However, global agricultural production faces pressures from climate change, resource scarcity, and rising nutritional demands. To adapt to changes in their environment, plants evolved precise and sophisticated gene expression regulatory mechanisms. A majority of gene expression regulatory elements are located in promoters and untranslated regions of mRNA. This review aims to elucidate how promoters and 5′ untranslated regions function in complex synergy to regulate gene expression in maize. We discuss the structural organization of these regulatory elements, from their basic components to their integrated roles in shaping plant gene expression. Particular emphasis is placed on their significant impact on maize biotechnology, including strategies for controlling, fine-tuning, and enhancing gene expression for crop improvement. With this review we wish to guide future biotechnological innovations and food security.

## 1. Introduction

Eukaryotic gene expression relies on conserved mechanisms involving *cis*-regulatory elements (CREs), chromatin architecture, and RNA processing. However, plants have evolved several distinct features that reflect their unique developmental needs and sessile lifestyle. For instance, plants possess specialized nuclear RNA polymerases, Pol IV and Pol V, dedicated to transcribing noncoding RNAs essential for RNA-directed DNA methylation (RdDM). Transcriptional silencing via the RdDM pathway is essential for maintaining genome stability in plants, which commonly contain transposable elements and repetitive sequences. Additionally, RdDM plays a role in pathogen defense, abiotic stress response, and the regulation of several key developmental transitions [1]. The functional diversity of the abundant long non-coding RNAs in plants expands the range of potential regulatory mechanisms by interacting with chromatin-modifying proteins or sequestering regulatory proteins, thereby modulating gene expression [2,3]. Likewise, RNA-binding proteins in plants have undergone extensive expansion and diversification relative to other eukaryotes, providing an additional layer of post-transcriptional regulation essential for adaptation to environmental stresses [4]. These evolutionary adaptations underscore the complexity of regulatory networks in plants.

Along with unique features, plants also possess the fundamental eukaryotic transcription regulators, including promoters and untranslated regions of mRNA. The promoter acts as the primary controller, determining the transcription initiation site and orchestrating the basal transcriptional machinery. Plant promoters consist of a core promoter which includes the transcription start site (TSS), proximal promoter elements, and distal regions. These elements serve as recognition sites for RNA polymerase II and associated transcription factors (TFs) binding to CREs, ensuring that transcription occurs at the correct time, in the proper cellular context, and with appropriate frequency [5]. Plant *cis*-regulatory landscapes are characterized by a strong promoter-proximal preference. For example, a high-resolution chromatin accessibility study has shown that the bulk of open chromatin regions are located within 3 kb upstream of TSSs. Such localization indicates that in plants gene expression is regulated more by proximal regulatory elements, rather than by distal ones, as in animals [6].

Another key regulator of gene expression in eukaryotes is the 5′ untranslated region of mRNA (5′UTR). Functionally distinct from promoters, 5′UTRs are located immediately downstream of the TSS and regulate gene expression post-transcriptionally. They can harbor various *cis*-acting elements, such as upstream open reading frames (uORFs), enhancers, introns and may form stable secondary structures including hairpins and G-quadruplexes. Altogether 5′UTR parts affect ribosome binding efficiency, scanning and initiation of translation, impacting protein synthesis rates [7,8].

One of the primary purposes of modern biotechnology is agricultural improvement, including abiotic and biotic stress tolerance increase and nutritional enhancement. Among the wide variety of model organisms, maize (*Zea mays*) appears to be the most suitable for solving biotechnological problems. High-calorie and protein-rich seeds make maize an important food for humans and an essential component of livestock feed. In addition to its nutritional value, maize also has significant industrial importance, especially for biofuel production [9]. However, maize production is increasingly threatened by abiotic stresses, with annual crop failure commonly attributed to weather extremes like drought and high heat [10]. In response, biotechnological advances have been directed toward enhancing key traits, including drought tolerance, agronomic performance, and grain quality [11]. The comprehension of basic mechanisms of gene expression regulation, governed mainly by promoters and 5′UTRs, underlies the aforementioned innovations. Although promoters and 5′UTRs operate at different stages of gene expression, they both control the spatial, temporal, and quantitative aspects of protein production [12]. This coordinated regulation at multiple levels is crucial for developmental processes, cellular differentiation, and responses to environmental signals [13]. This review focuses on the features of promoters and 5′UTRs, as well as their interactions, specifically for gene expression fine-tuning in maize, a well-studied monocot model. It synthesizes current knowledge spanning sequence-level characteristics, chromatin- and enhancer-level regulation, translational control, and machine-learning-guided approaches, highlighting the potential of these regulatory elements for agricultural improvement in maize.

## 2. Promoters

As the primary driver of transcription, the promoter retains an essential function across species, though its specific structure and regulation are frequently taxon-specific. In monocot, promoters tend to be GC-rich, as well as their coding sequences. In contrast, promoters of eudicots are usually AT-rich [14,15]. Also, GC-content bias in promoters can be observed between actively and rarely expressing genes, as high GC-content in promoters correlates with nucleosome depletion and facilitated transcription initiation [16]. Low functional compatibility between monocot and eudicot promoters was demonstrated in a cross-species study performed by Jores et al. [17]. In this work, AT-rich promoters from *Arabidopsis thaliana* activated transcription in tobacco protoplasts significantly more efficiently than in maize cells, as well as GC-rich promoters from maize performed better in maize cells.

### 2.1. Structural Features of Promoter Organization in Maize

**Figure 1:**
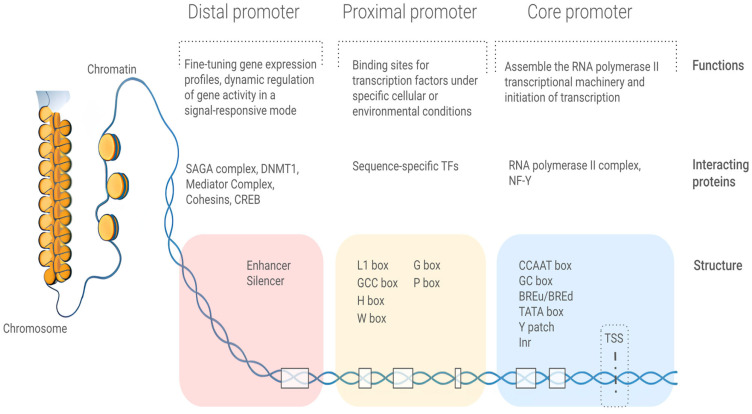
Hierarchical organization and functional architecture of plant promoters. The colored boxes represent distinct promoter parts. Abbreviations: BREu/BREd, upstream/downstream, B-recognition elements; CREB, cAMP Response Element-Binding protein; DNMT1, DNA (cytosine-5)-methyltransferase 1; Inr, initiator; NF-Y, Nuclear Factor Y; SAGA, Spt-Ada-Gcn5 Acetyltransferase; TFs, transcription factors.

Generally, maize promoters are operationally defined as the 1–2 kb region upstream of the transcription start site, including various regulatory features critical for transcriptional initiation (Figure 1) [18]. In common, maize promoter regions may be positionally and functionally divided into three parts: core promoter, proximal promoter, and distal promoter. Core promoters contain the most crucial elements for gene expression, including the TSS, the Inr (initiator), the Y patch, the TATA box, the CCAAT box, and others. These elements define the transcription start site and facilitate the recruitment of RNA polymerase II and associated transcriptional machinery. The proximal promoter harbors clustered CREs. Sequence-specific TFs bind to these CREs and act as key integrators of environmental signals (e.g., light, stress), enabling transcriptional reprogramming. The distal promoter contains enhancers, silencers, and insulators, which interact with core-bound transcriptional complexes through chromatin looping. They enable fine-tuning of spatiotemporal expression patterns by responding to hormones in developmental processes or tissue-specific signals through epigenetic modifications.

Das and Bansal analyzed promoter architecture features in several plant species, including maize [19]. For example, they suggested broadly expressed genes have less compact and less stable promoters in terms of free energy than tissue-specific promoters. In addition, promoter stability may be species-specific. Specifically, maize and other studied monocot promoters are more stable than those of *A. thaliana* in terms of their free energy. Also, maize promoters demonstrated higher DNase I sensitivity. DNase I sensitivity reflects variable nucleosome occupancy and chromatin remodeling dynamics, which determine the accessibility of promoters to tissue-specific or other regulatory factors [19].

#### 2.1.1. Core Promoter Region

As the name suggests, the transcription start site is the location of the first transcribed nucleotide. Based on the number and distribution of TSSs, core promoters can be divided into two distinct classes. Sharp promoters exhibit a highly focused TSS distribution with one or a few TSSs concentrated at a narrow range of nucleotide positions [20,21]. This precise architecture is typically supported by well-defined core motifs, such as the TATA box and Initiator sequences, positioned accurately relative to the TSS. Consequently, sharp promoters are often associated with tightly regulated, tissue-specific gene expression where precise initiation control is essential [22]. In contrast, broad promoters lack a single dominant TSS and instead harbor multiple closely spaced initiation sites. This structure commonly underlies the constitutive expression of housekeeping genes across diverse tissues [22]. In maize, the proportion of genes with sharp promoters ranges from 50% to 87%, contrasting with the approximately 36% observed in *A. thaliana* [23].

Core promoters often contain a TATA box, with a consensus sequence TATA(A/T)A(A/T). The main function of the TATA box is to bind TATA-binding protein (TBP), a subunit of TFIID. This interaction results in RNA polymerase II recruitment. The TATA box is required not only for transcription initiation but also to determine the level of gene expression in plants [24,25].

The location of core promoter elements relative to the TSS varies not only among different species, but also among the genes of the same species. Usually, it can only be described in probabilistic terms [26]. Thus, it is considered that the position of the TATA box in flowering plants is highly conserved – it is located mainly at −60 bp to −20 bp relative to TSS [14]. In maize promoters, the TATA box is located mainly at ~30 bp upstream of TSS with additional localization maxima at −55 and −70 [17]. At the same time, the distance between TATA-box and TSS negatively correlates with the strength of promoters, with maximal enhancement observed at −59 to −23 relative to the TSS [17].

Massive core promoter elements research revealed that TATA boxes are not universally present. Among 12,749 analyzed *Arabidopsis thaliana* (dicot) genes, merely ~29% contained a TATA box motif [27]. In monocot genomes TATA box was observed in ~19% *Oryza sativa* [28] and in 19–38% *Zea mays* promoters [17,23,26]. In addition, genes with broad promoters in maize are significantly less likely to contain a TATA box compared to sharp promoters [23]. Interestingly, maize promoters with TATA box demonstrated up to 4-fold greater transcriptional activity than TATA-less analogs [17].

Another important element of the plant core promoter is the Initiator sequence (Inr), which also initiates transcription by binding to general transcription factors and RNA polymerase II. Inr is a short DNA sequence that typically overlaps or lies very close to the TSS. Although Inr functionality is independent from the TATA box, it can also act synergistically [21]. For instance, light-mediated expression requires both Inr and the TATA box simultaneously [29]. Like the TATA box, Inr sequences are relatively widely distributed in the genes, with patterns of distribution varying among plant groups [26]. Genome-wide computational promoter elements analysis has predicted positional distribution of Inr in eudicots in the range −20 bp to +240 bp, followed by a gradual decrease in frequency with a maximum at −15 bp relative to TSS [14]. In monocots, this study described a more normal distribution of Inr frequencies in the range from approximately −170 bp to +250 bp, with a maximum also at −15 bp relative to TSS [14]. It is important to note that this broad distribution likely reflects a range of Inr-like signals present in the promoter and 5′ untranslated region rather than the true Inr motif used by the transcriptional machinery.

Along with the eukaryotic common core promoter elements, plants have unique ones. One such unique element is the Y patch (or TC motif), named for the pyrimidine abundance [30,31]. The significant proportion of TATA-less core promoters in plants assumes the presence of functional compensation by other promoter elements, such as the Initiator and Y patch motifs. Synthetic promoter screening confirmed that the Y patch motif can boost promoter strength up to 2-fold, while wild-type Y patch-containing promoters also demonstrated 10–15% greater strength compared to Y patch-less [17]. Although the most effective promoters in Jores et al. work contained the TATA box, Inr and Y patch simultaneously, the presence of the TATA box provided the strongest effect on promoter strength [17]. Also, Sanchez-Muñoz et al. demonstrated methylation accumulation for the Y patch, suggesting its potential role in methylation-dependent gene expression regulation [32].

The predicted distribution of Y patch motifs in eudicot and monocot promoters is similar, from −30 bp to +250 bp relative to TSS [14]. Approximately 50% of rice (*Oryza sativa*) core promoters contain one or more Y-patches, suggesting this element represents a functionally significant *cis*-regulatory feature [28].

In plant core promoters, the TATA box may be flanked by the TFIIB recognition element located upstream (BREu) or downstream (BREd). In eukaryotic cells, BREs modulate gene expression through interaction with TFIIB [33,34]. Although the details of BRE functioning in plants remain poorly characterized, BREu has been shown to enhance the strength of synthetic promoters in maize protoplasts by approximately 25%, whereas BREd elements reduce strength by ~10% [17]. Since this effect was not observed in tobacco leaves, a species-specific regulation through the maize TFIIB ortholog can be suggested [17].

The CCAAT box, defined by the CCAAT consensus sequence, is another common motif in the core promoter region, mainly positioned from −120 bp to −40 bp in eudicots and −460 bp to −140 bp in monocots, including maize [14]. This motif is the binding site for the nuclear factor Y (NF-Y) complex-a heterotrimeric transcription factor that participates in chromatin remodeling [35]. After binding, NF-Y can provoke both positive and negative histone modifications through specific enzymes [36,37], thus, positively or negatively affecting gene expression [35]. Despite there is a huge knowledge gap about the CCAAT motif and NF-Y functional roles in plants, research is currently being extensively conducted. For example, Xiong et al. demonstrated the abundance of CCAAT motifs in storage protein genes of rice, associating starch formation with NF-Y regulation [38].

Another common eukaryotic core promoter element is the GC box with a consensus sequence GGGCGG. In plant genes, the GC box is often found in multiple copies on both DNA strands within a broad range from −70 to +205 relative to TSS [14]. GC box hexamers are not found in plant promoters as often as in mammalian ones. Approximately 25% of maize promoters and only 7% of Arabidopsis promoters contain at least one copy of this motif [26]. In animal systems, the GC box interacts with several transcription factors, including Sp1 and other proteins of the Sp family, to enhance transcription rate significantly [39,40]. However, to the best of our knowledge, no association between Sp1 and plant promoter was found as well as Sp1 protein homologs in *Arabidopsis thaliana* and *Oryza sativa* genomes [41], suggesting the presence of alternative GC box-interacting proteins.

Yet another poorly characterized plant core promoter element is the Downstream Promoter Element (DPE). It was found and well characterized in *Drosophila* [42] and later described as conserved from fruit fly to humans [43]. In animals, it is often associated with TATA-less promoters and spaced precisely from Inr, which is important for TFIID binding [43]. Despite the predicted distribution of DPEs in plant promoters [14] and occasional mentions in review articles [33,44], no direct evidence of participation of these elements in transcription regulation in the plant cells was found.

Despite the cruciality of core promoter elements for gene expression, transcription could be regulated by coreless promoters. Such relatively short promoters lack traditional core elements such as TATA and Y patch. They are usually located far upstream of the TSS and associated with low-expression genes [45]. In eudicots, coreless promoters are often found in uniformly expressed genes. In contrast, monocot species tend to have a strong association between coreless promoters and conditionally expressed genes [46].

#### 2.1.2. Proximal and Distal Promoter Regions

In contrast to well-characterized and highly conserved core promoters, proximal and distal promoter parts are more variable across different species and tissues. The proximal promoter region is located several hundred bp upstream of the TSS and harbors a diverse set of *cis*-regulatory elements and motifs that determine promoter strength, tissue specificity, and ability to respond to both developmental and environmental signals [16].

Typically, proximal promoters contain diverse *cis*-regulatory elements rather than uniform motifs. For example, CRE sequences and corresponding transcription factors involved in abiotic stress responses have been well studied [47]. Another group of CREs that interact with the MYB (myeloblastosis) transcription factor family plays a crucial role in plant development and defense responses. They regulate such important processes for viability as the cell cycle control, cell differentiation, the central circadian oscillator, secondary metabolism, and the regulation of stress signaling [48]. The W box element is a key target for WRKY transcription factors and often serves as a regulator of seed germination, pollen development, and hormonal regulation [49]. MCM1/AGAMOUS/DEFICIENS/SRF (MADS) box-interacting transcription factors play a role in the regulation of the entire plant life cycle through developmental processes control, including seed germination, vegetative growth, transition from vegetative to reproductive growth, floral development, and senescence, and additionally regulating the abiotic and biotic stress tolerance [50]. Also, some CREs are involved in hormone signaling. Key examples include the abscisic acid-responsive element (ABRE), the TGACG motif recognized by methyl jasmonate-responsive factors, the ethylene-responsive GCC box, and the salicylic acid-responsive TCA element [51].

Mapping CRE variants and their interactions with TFs under various stimuli is an active area of research in both eudicots and monocots [50,51,52]. For practical use, special databases have been established describing hundreds of diverse CREs and TFs found in flowering plants [53,54,55]. Several maize proximal promoters have also been thoroughly investigated in search for regulatory elements. For instance, the β-carotene hydroxylase 2 (ZmBCH2) promoter demonstrates the contribution of specific *cis*-elements, such as the P box and AACA motif, to seed-specific transcriptional activation through interactions with ZmPBF and ZmGAMYB transcription factors [56].

Finally, the most distant from the TSS promoter part, the distal promoter region is located at kilobases upstream or downstream of the core promoter. This region harbors *cis*-regulatory modules (CRMs), which in contrast to individual transcription factor binding sites containing CREs, represent a higher-order organization. CRMs include such functional units as enhancers, silencers, and insulators in distal promoter regions.

Enhancers are CRMs that promote transcription by recruiting specific TFs and cofactors to precisely increase the initiation rate of target genes in a tissue-, time-, or signal-specific manner [57]. Recent findings indicate that enhancers in plants act depending on their specific position relative to the TSS, contrasting with the position-independent nature seen in animals and yeast, highlighting a key difference in transcriptional regulation [58]. The molecular mechanisms by which enhancers regulate gene expression are complex and beyond the scope of this review. Several other reviews provide more detailed information on current known mechanisms [57,59,60]. Importantly, enhancers often participate in the formation of chromatin loops, establishing direct spatial interactions with core promoters to regulate transcription. Such enhancer-promoter loops have been observed in plants with moderate to large genomes, including rice, maize, and wheat [61]. Thus, genome-wide analysis identified approximately 1500 intergenic enhancer candidates and defined their tissue-specificity [62]. As revealed by Hi-C and HiChIP analyses, these loops in maize frequently exceed 20 kb in length, suggesting the crucial role of long-range CREs in maize gene regulation [63]. For example, several well-characterized maize enhancers located in kilobases from the transcription site they regulate [64,65,66].

As functional antagonists to enhancers, silencers could silence a gene directly, but also indirectly by disrupting enhancer-promoter communication and preventing transcriptional activation. Genome-wide analysis has identified silencing-associated chromatin signatures. In maize, for example, chromatin loops associated with H3K27me3-marked nucleosomes exhibited lower transcript levels than those associated with H3K4me3 [63].

The last large group of regulatory elements in distal promoters is insulators. An insulator is a DNA element that prevents the action of CRMs on a promoter. It is usually located between a target CRM and the core promoter and capable of binding specific proteins, blocking gene activation or silencing. Several studies suggest the presence of enhancer-blocking insulator sequences in plants [67,68,69]. However, the existence of true insulator elements in plants remains highly debated. Previous reviews have concluded that CRMs with definitive insulator function have not been identified, raising doubts about their presence in plant genomes [57,61]. This uncertainty is amplified by the absence of the most studied metazoan insulator protein—CCCTC-binding factor, suggesting convergent evolution of one or more functionally equivalent chromatin architectural regulators in plants [61]. Adding to this discussion, a recent study has identified over 100 different 170 bp fragments active as insulators with different enhancers and promoters across diverse plant tissues [70]. Intriguingly, these elements exhibited bifunctional activity: they behaved as silencers when paired with weak enhancers but functioned as insulators against strong enhancers. This context-dependent behavior suggests regulation that depends on the local environment rather than on canonical insulators, indicating a potentially uncharacterized form of *cis*-regulatory logic [70]. Thus, while the debate continues, these new insights offer a fresh perspective on this complex topic.

The remarkable morphological and functional diversity of plant tissues is the result of precise spatiotemporal gene regulation managed by the regulatory network of CREs and CRMs. Within these networks, enhancers and silencers work together to precisely balance gene expression and create boundaries between different cell types to express or not a target gene [71]. Lewis et al. suggested that complex expression patterns evolved by integrating repressor sites to silence broadly active enhancers in specific cell types [71]. Moreover, further complexity to understanding arises from the observation that a significant fraction of distal putative CRMs in maize did not interact with their immediately flanking genes, but instead bypassed them by interacting with more distal genes [72]. Therefore, multi-tissue maps of chromatin accessibility are extremely useful for deciphering this *cis*-regulatory logic in maize [73,74,75,76,77].

### 2.2. Applications of Promoter Diversity for Maize Genetic Engineering

The identification and functional validation of promoters represent an important objective in plant biotechnology. Although extensive research has characterized and exploited various promoters in model eudicot, their functional equivalence in monocot crops cannot be assumed. Substantial differences in core promoter architecture, transcription factor repertoires, and epigenetic landscapes between these major plant clades often lead to differing transcriptional behaviors. TF binding sites in proximal promoter regions can be associated with altered promoter strength in one species but not in another, reflecting species-specific regulatory network differences [17]. In practice, it means that strong eudicotyledon promoters, such as Cauliflower mosaic virus 35S promoter (CaMV 35S), may show weaker activity in monocots [78]. And vice versa: strong in monocots, maize ubiquitin-1 promoter (ZmUbi1) and the rice actin-1 promoter (OsAct1) are much less effective in eudicot tissues [78,79]. Here, we consider several different classes of promoters that are widely used in plant biotechnology. Also, we will try to identify several principles of choice specific promoters suitable for different tasks.

#### 2.2.1. Constitutive Promoters

The most widely used promoters in the plant biotechnology are constitutive promoters, which drive gene expression continuously in most or all tissues throughout the life of the plant, independent of developmental stage or environmental signals. Constitutive promoters are often associated with genes essential for maintaining basic cellular functions. Absence of specific regulatory elements allows one not to worry that these promoters will behave differently in different tissues or will not work at all. Even a small fraction of possible constitutive promoters with different strengths, such as ubiquitins (ZmUbi1, PGNpr1, rubi3), H2B (maize H2B promoter), and Gos-2 (ZmGOS2) [80,81,82,83,84,85], allows researchers to create a precise toolkit for advanced genetic engineering of maize (Table 1).

**Table 1:** Functionally validated promoters with demonstrated activity in maize.

References	Specification	Promoter	Type
[80,81,82,83,84,85]	constitutive	ZmUbi1, PGNpr1, rubi3, H2B, Gos2	Plant constitutive
[86,87,88]	constitutive	CaMV 35S, FMV, CmYLCV, CsVMV	Viral constitutive
[89]	embryo and leaves	Zm-PLTP	Tissue-specific
[90]	embryo	OLE, EAP1, LTP2	
[91]	root	p8463, p5023, p1534, p8531, p6629	
[92]	endosperm	LWM	
[93]	embryo	globulin-1	
[94]	silk tissues	p1-R2R3-MYB	
[95]	pollen	ZmSTK2_USP	
[96]	wound-inducible	MPI	Inducible
[97]	senescence-inducible	Zm(PSEE1)	
[89]	auxin-inducible	Zm-Axig1	
[98]	drought and salinity	ZmGAPP	
[99]	abscisic acid-inducible	ABA-inducible	
[100]	chimeric	superpromoter	Synthetic
[101]	chimeric	A27znGlb1	
[102]	synthetic abscisic acid-response	ZmDRO1	
[103]	bidirectional	BDP	

A huge group of constitutive promoters that are currently in biotechnological and scientific use are promoters derived from plant viruses. Multiple caulimoviridae members, including Cauliflower mosaic virus (CaMV 35S), Figwort mosaic virus (FMV) [86], Cestrum yellow leaf curling virus (CmYLCV) [87], and Cassava vein mosaic virus (CsVMV) [88], have been characterized and drive strong heterologous expression in transgenic maize. Notably, the CmYLCV promoter has been shown to outperform both the CaMV 35S and ZmUbi1 promoters by 2-fold in maize [87].

Cereal engineering presents distinct challenges, as many synthetic biology tools were developed for model eudicot species. Currently, it relies on a limited set of constitutive promoters, with transgene expression further modulated by codon optimization, intron-mediated enhancement, and terminator selection. To address this limitation and expand the repertoire of effective regulatory elements, a wide comparison of diverse promoters was conducted to identify optimal regulatory elements for precise and robust transgene expression in cereal engineering [104]. However, empirical validation remains necessary for orthologous promoters due to their potential for variable expression fidelity [105], even though they are derived from phylogenetically related monocots. As an example, the rice OsAct1 promoter drives strong expression in maize [106], providing cross-species functionality. New work using maize protoplasts addresses the lack of standardized regulatory elements for synthetic biology in cereals. Also, in a recent article, May et al. characterized a diverse set of gene expression elements, including 17 promoters and 18 promoter-5′UTR fusions [107]. Thus, this study provided essential engineering components and critical insights into the cross-species functionality of promoters in maize [107].

#### 2.2.2. Tissue-Specific and Inducible Promoters

Some tasks require gene expression activation in specific tissues or upon external chemical or physical signals. For example, application of tissue-specific promoters in transgenic engineering is particularly valuable when precise targeting of metabolic pathways or developmental processes is required, as it allows for enhanced trait performance without compromising plant growth. The functionality of this approach is illustrated by the maize promoter Zm-PLTP that drives gene expression specifically in embryos and leaves while showing limited expression in the ear, tassel, and roots. The expression of the morphogenic gene *Bbm* under Zm-PLTP control provided rapid and uniform formation of somatic embryos directly from the scutellum, leading to efficient plant regeneration without the pleiotropic effects associated with constitutive expression [89].

Seed-preferred promoters are the most extensively characterized class of tissue-specific regulatory elements, primarily used for metabolic engineering of grain nutritional profiles and seed oil biosynthesis [105]. Their application extends to recombinant protein accumulation, capitalizing on the seed endogenous biochemical environment, optimized for long-term macromolecular stability and storage. For example, embryo-specific promoters, including an oleosin (OLE) promoter, an early embryo protein (EAP1) promoter, and an aleurone-specific lipid transfer protein (LTP2) promoter, were used to achieve a high-level expression of two key TFs involved in the regulation of oil accumulation in maize. This approach substantially elevated seed oil content, demonstrating how tissue-engineered expression systems can optimize metabolic traits [90]. Five promoters driving root-predominant gene expression in maize were characterized, which could potentially lead to improved root architecture [91]. Additional maize-derived tissue-specific promoters could provide enhanced spatiotemporal precision by leveraging native regulation: LMW (low molecular weight) glutenin promoter in endosperm [92], globulin-1 promoter in embryo [93], p1-R2R3-MYB in silk tissues [94], and ZmSTK2_USP pollen-specific [95].

While tissue-specific promoters offer spatial control, restricting expression to defined anatomical regions or cell types, inducible promoters are designed to provide temporal control over gene expression by responding to specific exogenous stimuli or developmental signals. This conditional expression strategy minimizes detrimental effects associated with continuous expression. In maize, inducible promoters have been developed to respond to wound, senescence, and environmental stimuli such as drought, heat shock, and chemical inducers. Specifically, the maize proteinase inhibitor (MPI) promoter exhibits wound-inducible properties, effectively driving the expression of target genes in response to mechanical damage or pest attack. In nature, this inducibility allows the plant to locally increase the production of defensive proteins upon wounding, thereby enhancing resistance against herbivory or pathogen invasion. Studies have documented that the MPI promoter can yield significant expression of insecticidal proteins like Cry1B, albeit with a slight lag in expression relative to constitutive promoters [96]. Additionally, there are several inducible promoters with different inducing stimuli that have also been characterized in maize: senescence-Inducible promoter ZmP(SEE1) [97], auxin-Inducible promoter Zm-Axig1 [89], drought and salinity inducible promoter ZmGAPP [98], abscisic acid-inducible promoter [99].

#### 2.2.3. Synthetic Promoters

Enhancing promoter strength is a main strategy for achieving higher transgene expression in plants. This can be pursued through multiple ways, including the discovery of novel natural promoters and the design of synthetic ones. Natural promoters offer the advantage of evolutionary conservation and a well-established track record in their native regulatory roles; however they may be limited by moderate expression strength, size, and issues related to homology-based silencing [108]. In some cases, such as the use of pathogen-derived promoters like the CaMV 35S, this can trigger unintended phenotypes or gene silencing due to sequence repetition [109]. Also, synthetic promoters can provide enhanced flexibility and the ability to fine-tune expression patterns, ranging from spatial and temporal specificity to inducibility and bidirectionality. However, their application demands sophisticated design strategies and rigorous validation to ensure predictable performance [110,111]. Moreover, synthetic promoters also have the advantage of reduced sequence length, which facilitates easier vector construction and diminishes the likelihood of homology-dependent gene silencing in complex genetic constructions [112].

Current synthetic promoter design efforts remain focused on model plants with the most studied regulatory architectures, leaving maize relatively underexplored. A significant obstacle in maize synthetic promoter development is the incomplete understanding of functional interplay among diverse CREs [18,109]. Despite these challenges, researchers have created a number of synthetic promoters in maize. For example, a chimeric ‘superpromoter’ was engineered through the fusion of three copies of the octopine synthase transcriptional activator with the mannopine synthase (*mas20*) activator and minimal promoter [100]. In transgenic maize systems, this hybrid construct exhibited transcriptional activity comparable to or exceeding established viral (CaMV 35S) and endogenous (ZmUbi1) constitutive promoters [100]. The fusion of regulatory sequences from the maize 27zn and Glb1 promoters yielded the synthetic chimeric promoter A27znGlb1. This engineered promoter demonstrated tissue-specific expression, directing transgene activity specifically within the embryo and endosperm of transgenic maize plants [101]. In another study, a synthetically engineered ABA-responsive promoter directed *ZmDRO1* expression in maize, conferring steeper root growth angles and enhanced drought tolerance [102].

Careful modification engineering of natural promoters enables the creation of synthetic regulatory variants with fine-tuned functionality. Illustrating this approach, researchers used CRISPR-Cas9 to generate weak allelic variants of key maize CLE gene promoters. These edits achieved quantitative reduction in target gene expression without perturbing overall plant architecture, ultimately conferring enlarged ears, increased kernel rows, and higher grain yield per ear [113].

Many plant traits are polygenic, making phenotype modification more complicated. To mitigate gene silencing in multigene cassettes and reduce the size of transferred DNA, bidirectional promoters and gene stacking strategies can be employed [114]. A synthetic cassette incorporating a bidirectional promoter derived from the ZmUbi1 promoter, combined with a bicistronic gene arrangement, enabled the coordinated expression of four genes with higher efficiency compared to the expression of a single gene driven by the ZmUbi1 promoter alone in maize [103]. Additionally, for metabolic engineering of anthocyanin-rich maize, researchers have successfully utilized a seed-specific bidirectional promoter [115].

However, in case of difficulties in synthetic bidirectional promoter application including legislative restrictions, there are several natural promoters of this kind. For example, the intergenic region between maize *Def1* and *Def2* defensin-like genes is a naturally evolved bidirectional promoter. Functional analyses in immature embryos revealed that this compact (~635 bp) element drives asymmetric co-expression of both genes, proving that even minimal promoter sequences can orchestrate sophisticated spatiotemporal and directional control programs [116].

Finally, one could combine synthetic promoters with synthetic transcription factors to control gene expression more precisely. For instance, synthetic zinc finger TFs have demonstrated functional activity in maize protoplasts [117].

Despite these advantages, synthetic regulatory elements have several limitations. The design process for these elements often involves alternating extensive bioinformatics analysis and experimental validation to achieve the desired functional characteristics [112]. Incorrect combination of *cis*-elements or positioning can lead to unwanted transcription factor competition or steric hindrance, resulting in unpredictable expression levels. Moreover, synthetic promoters may not fully reproduce the nuances of adaptive responses of natural regulatory networks, especially under changing physiological conditions [112].

## 3. 5′ Untranslated Regions

The term “promoter” is commonly used to describe the whole regulatory region upstream of the start codon, including the promoter itself and the 5′ untranslated region (5′UTR). Whilst the promoter orchestrates transcription initiation and affects the number of transcripts, the 5′UTR plays a crucial role in determination of translation efficiency. As an example, several synthetic “promoters” used in maize include the 5′UTR intron of genes encoding Ubiquitin-1/2 or Adh1 for intron-mediated enhancement. In contrast, use of a poorly optimized 5′UTR could lead to lower protein expression due to inefficient translation [118,119]. Thus, to achieve the desired protein production level, one should improve both transcript quantity and quality [120]. Therefore, the development of plant synthetic promoters or optimization of existing ones is typically accompanied by the selection and refining of an appropriate 5′UTR sequence for translation.

### 3.1. The Functional Roles of the 5′UTR

**Figure 2:**
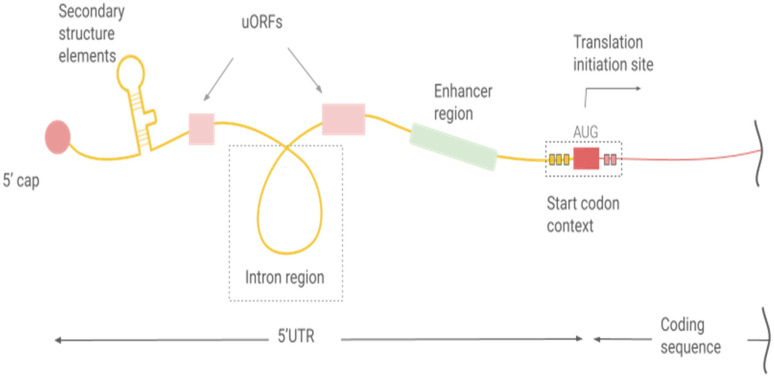
Schematic representation of regulatory elements within the 5′ untranslated region (5′UTR). The composition, quantity, and position in sequence of elements may vary among different 5′UTRs.

The 5′UTR of an mRNA is a non-coding leader sequence between the transcription start site and the translation start site (start codon). The 5′UTR, together with the 3′UTR, is recognized as a key regulatory hub for post-transcriptional control [121]. Though not translated, it profoundly influences translation initiation and mRNA stability. Key functional features within the 5′UTR include the 5′ cap structure that recruits initiation factor eIF4E for translation initiation, regulatory elements modulating ribosomal scanning (e.g., stem-loops or upstream ORFs), embedded *cis*-acting elements such as translation enhancers and intronic sequences, and the start codon context sequence (Figure 2).

The composition and arrangement of 5′UTR regulatory elements and mRNA secondary structures regulate the susceptibility of mRNA to ribonuclease-mediated degradation, determining mRNA half-life and availability for translation [5,122]. By modulating both the initiation of translation and the stability of the mRNA, 5′UTRs provide a fine-tuning mechanism that allows cells to rapidly adjust protein output in response to changing physiological conditions without alterations in transcription rates [123].

In monocots, the functional significance of the 5′UTR extends to the regulation of gene expression under stress conditions such as drought, heat shock, and nutrient deficiency. For example, the splicing efficiency of 5′UTR introns in maize is sensitive to heat shock, resulting in increased expression of ubiquitin proteins [124]. Another functional significance of monocot 5′UTRs in stress-responsive gene expression is highlighted by the maize alcohol dehydrogenase (*adh1*) 5′UTR, which drives efficient translation under hypoxia and heat shock conditions. Incorporation of this 5′UTR into expression cassettes provided efficient target protein production in plants under stress conditions [125].

The length and content of 5′UTRs in plants correlate with the functional role of the gene [7,126]. Functional enrichment analysis in Arabidopsis and rice revealed functional differences between 5′UTRs of different lengths [127]. Similar analysis in maize suggested that genes related to developmental processes, response to abiotic stimuli, organic substance transport, anatomical structure development, and mRNA transport possess short 5′UTRs (1–500 bp). Medium-length 5′UTRs (501–1000 bp) are associated with genes involved in phosphorus metabolic processes, protein phosphorylation, cellular protein modification processes, cell communication, and the regulation of cellular processes. Genes associated with the regulation of iron ion transport were enriched across both the long (1001–2000 bp) and very long (>2000 bp) 5′UTR length categories [128].

In maize, 5′ and 3′UTR sequences make up approximately 24.7% of the total transcripts length. The average maize 5′UTR length is 430 bp, longer than that observed in rice (259 bp) and Arabidopsis (155 bp) [128]. Sometimes the number of *cis*-acting elements in 5′UTR is positively correlated with the expression level of the corresponding gene. This was revealed, for example, for the *Zea mays* LAZ1 gene family. Furthermore, 5′ UTRs of the ZmLAZ1 gene family not only modulate gene expression but also impart functional specificity in a length-dependent manner [128]. These results suggest the important role of 5′UTR architecture in gene regulation and functional diversification of gene expression in maize.

### 3.2. 5′ Capping

The 5′ cap is a characteristic structure of eukaryotic mRNAs. In maize, as in other eukaryotes, the canonical cap consists of a 7-methylated guanosine (m7G) linked by a 5′,5′-triphosphate chain to the first transcribed nucleotide at the very 5′ end of nascent transcripts, thereby protecting the mRNA from exonucleolytic decay [129,130]. There are several types of caps: Cap-0 (m^7^Gppp), Cap-1 (m^7^GpppN^1^_m_), and Cap-2 (m^7^GpppN^1^_m_N^2^_m_), where N_m_ is a 2′-O-methylated nucleotide and ppp indicates a triphosphate bridge. Mammalian mRNA typically possesses the Cap-1 and Cap-2 structures, whereas mRNAs with the Cap-0 are recognized as extrinsic RNA, triggering the immune response. However, elevated level of Cap-1-containing transcripts could still induce an innate immune response, albeit weaker [131,132]. Recent research has shown that the mRNA of arabidopsis and maize do not contain the Cap-1 or Cap-2 forms. This suggests a different mechanism and function of mRNA capping in gene regulation in plants [133].

The 5′ cap mediates cap-dependent translation by recruiting the eukaryotic initiation factor complex eIF4F through 5′ cap-eIF4E interaction [134]. This interaction is central to the assembly of the 43S preinitiation complex that scans the mRNA for the start codon. Specifically, in maize, selective cap-binding by isoforms such as eIF4E and eIF(iso)4E is critical during seed germination. Dynamic changes in the composition of cap-binding complexes modulate the selective translation of stored mRNAs and *de novo* transcripts [135,136].

Under stress conditions, such as heat shock, viral infection, or apoptosis, the efficiency of cap-dependent translation can be drastically reduced [137]. In response to stress, plants may use an alternative mechanism of translation initiation using internal ribosome entry site (IRES) mediated mRNA translation to maintain the synthesis of essential proteins. IRES is a sequence of mRNA with a set of *cis*-regulatory elements that can directly recruit the ribosomal subunits to the mRNA or interact with IRES *trans*-acting factors (ITAFs) for ribosome recruitment in eukaryotic cells [138]. While the mechanisms of IRES-dependent initiation have been well-characterized in animals, they are still not well understood in plants, with only a few examples available. For instance, *A. thaliana* mRNA translation of the WUSCHEL transcription factor, which plays a central role in maintaining and regulating stem cell populations in shoot and floral meristems, is IRES-mediated and is enhanced by environmental stress [139]. Scarce IRES data are available in maize, with one well-characterized example being the 5′UTR of *hsp101* mRNA, which contains an IRES that facilitates internal ribosome recruitment and translation initiation even when cap-dependent pathways are blocked. This guarantees the production of a critical cell thermotolerance chaperone protein [129].

In addition to the common m7G 5′ caps, several plant studies have reported a few more cap modifications. Non-canonical caps such as NAD+ have been suggested as important regulators of RNA fate through their influence on mRNA stability and translation. For instance, the Arabidopsis *dxo1* mutant, which lacks the NAD+ decapping enzyme DXO1, exhibits pleiotropic growth defects throughout its life cycle. This includes a dwarfing, light green coloration indicating total chlorophyll deficiency, and decreased fertility, giving smaller seed sets [140]. Advanced techniques, including NAD captureSeq, NAD-seq, and NAD tagSeq, have been successfully applied to map these modifications across plant transcriptomes. However, data on NAD+ caps in maize remain absent, pointing to a potentially significant and unexplored regulatory layer.

### 3.3. uORFs

Many plant 5′UTRs contain upstream open reading frames (uORFs) – start codons upstream of the main ORF. Genome-wide analysis shows that uORFs are widespread in a vast majority of eukaryotic genomes and probably have regulatory functions [141]. uORFs modulate translation by reducing ribosome reinitiation efficiency at the main ORF through ribosomal dissociation or stalling at the uORF and thereby preventing ribosomes from accessing the main open reading frame [142]. Recent research in *A. thaliana* has demonstrated that uORF length directly dictates translation reinitiation efficiency, with longer uORFs resulting in dramatically lower rates: ~95–100% translation reinitiation efficiency for a 3 aa uORF, declining to ~61% for 25 aa, ~25% for 40 aa, ~4% for 50 aa, and merely ~2% for a 70 aa uORF [143]. A small subset of uORFs, termed Conserved Peptide uORFs (CPuORFs), encode evolutionarily conserved peptides that are important for translational repression. For instance, a CPuORF within the *bZIP11* gene in Arabidopsis can prevent the ribosome from producing the main protein when sucrose levels are high, thereby adjusting gene expression accordingly [144]. There is a wealth of evidence from mutagenesis and ribosome profiling that CPuORFs stall the ribosome in a sequence-dependent manner [145]. Notably, many of these CPuORFs are conserved between monocots and eudicots [146].

The distribution of uORFs across genes is functionally biased. Highly expressed mRNAs, like those encoding housekeeping genes, typically possess short 5′UTRs lacking uORFs. Conversely, poorly expressed mRNAs, such as those for TFs and kinases, often feature longer 5′UTRs enriched with uORFs [146]. About 35% of *A. thaliana* genes are transcribed into uORF-containing mRNAs, and about half of these have multiple uORFs [147].

In maize, uORFs have been identified in genes encoding transcription factors, such as *Opaque-2* and other regulatory proteins, where overlapping uORFs exhibit redundant repressive functions [148]. These uORFs collectively restrict translation initiation of the main coding sequence unless specific cellular conditions, such as drought, are met. Additionally, in maize under drought stress, translation of 3063 uORFs associated with 2558 genes was detected, revealing a pervasive layer of translational control. This uORF-driven translational reprogramming functions synergistically with transcriptional regulation in the maize drought response [149]. Additionally, nucleotide variations near uORF start codons modulate translation efficiency by affecting the initiation process. This leads to inheritable allelic differences in protein levels that are associated with phenotypic traits and could serve as an adaptive function [150].

Mutagenesis of the uORFs’ start codons of the native 5′UTR can prevent their translation initiation. This strategy has been demonstrated to enhance translation efficiency of the primary open reading frame. For example, targeted disruption of an inhibitory uORF within the 5′UTR of the maize *Lc* transcription factor gene resulted in increased translation of the main ORF, correlating with elevated anthocyanin production [151]. Therefore, while designing genetic constructions, removing uORFs or avoiding introducing them inadvertently may be important for maximizing expression.

### 3.4. RNA Secondary Structure

mRNA molecules can form various secondary structures determined by intramolecular base pairing, yielding stable structures such as hairpins, stem-loops, pseudoknots, and G-quadruplexes. These structures are known to affect the accessibility to functionally significant mRNA regions, including the 5′ and 3′ untranslated regions as well as specific internal sequences in the coding region [152,153]. The tendency to form secondary structures can be estimated by mRNA free energy calculation, which is calculated from nucleotide sequence, taking into account the influence of the environment. Thus, changes in the cell environment (e.g., temperature or osmolarity) could affect mRNA secondary structures composition, making them an embedded transmitter of physical signals to the translation regulatory machinery. In particular, it has been suggested that plants have adopted the RNA G-quadruplex structure as an adaptation to cold conditions during evolution [154]. Temperature-sensitive RNA structural elements, known as RNA thermometers, also represent a regulatory mechanism in plants, where they modulate translational activity in response to thermal changes [155]. The secondary structure within the 5′UTR can affect the initiation of translation at AUG codons, especially in the case of weak start codon context [156,157].

Several studies demonstrated a role of the 5′UTR in translation repression in maize due to inefficient reinitiation of translation caused by upstream open reading frames and mRNA secondary structures [158,159,160]. Another study focused on maize 5′UTRs of hypoxia-induced genes distinguished two groups of untranslated regions based on the presence of stable secondary structures. The first group included relatively long 5′UTRs, which in common form stable secondary structures that may positionally coordinate translation initiation sites. In contrast, short 5′UTRs generally lack stable folding, suggesting distinct regulatory mechanisms of translational control [161].

Direct experimental validation of stable mRNA structures the impact on gene expression in maize remains limited. However, functional similarities across monocots allow one to project data obtained on other plants. In particular, this type of 5′UTR feature is relatively well investigated in *Oryza sativa*, making rice a model organism in the area of mRNA structure-function relationships in monocots. Thus, a study of the 5′UTR of the rice *OsMac1* gene identified several stem-loop structures, whose deletion sharply reduced translation efficiency [162]. Although this study focused primarily on translation, the necessity of a defined secondary structure for efficient protein synthesis implies that these features could also contribute to mRNA stability. Additionally, *in vivo* structural study showed total transcript unfolding upon heat shock, promoting transcript degradation [163].

High GC content in the 5′UTR can affect translation initiation efficiency by forming stable structures. This obstructs mRNA scanning by the 43S preinitiation complex, thereby modulating start codon accessibility and hindering completion of initiation [150]. The impact of GC content on the structure and functioning of RNA is also well-studied in rice. A study on translation in rice shows that high GC content of transcripts is correlated with high translation efficiency [164]. However, the *in vivo* study in rice has revealed that GC content has little impact on RNA structure formation in coding sequences, but strongly affects the RNA structure in untranslated regions, especially in 5′UTR [165]. A similar effect was revealed for wheat, where mRNA structure has a strong impact on translation, independent of GC content [166]. Additionally, mRNAs with regulatory functions tend to fold into more thermostable structures in rice [165].

### 3.5. Introns

5′ untranslated regions can also harbor intronic sequences. A 5′UTR intron, located within the segment of the primary transcript (pre-mRNA), is classified as an intron because it is transcribed as part of the pre-mRNA and can be subsequently excised by the spliceosome, similar to introns within the coding sequence (CDS) [5]. 5′UTR introns can exert significant regulatory effects, influencing mRNA nuclear export, translational efficiency, and mRNA stability [167,168,169]. The positive impact of introns on gene expression is termed intron-mediated enhancement (IME). For IME, introns usually should be located near the transcription start site. So they facilitate enhanced pre-mRNA processing, including efficient splicing and proper nuclear export, ultimately leading to higher levels of mature transcripts available for translation [170]. However, despite thorough research, the exact mechanism of IME remains unclear and has been explained by several distinct hypotheses. A particularly well-analyzed one proposes that introns stimulate transcript initiation by creating a favorable local chromatin structure [171]. Additionally, 5′UTR introns can possibly have an impact on transcription efficiency. An analysis of the rice Rubi3 5′UTR intron revealed its capacity to enhance both transcription (2-fold in nuclear run-on experiment) and mRNA accumulation (20-fold), supporting dual regulatory mechanisms while indicating post-transcriptional enhancement as primary [172].

In monocots, some researchers suggest intron-mediated enhancement often depends on splicing with a few exceptions [170]. 5′UTR introns have been used for protein production increase for quite a long time. The rice 5′UTR intron from the *salT* gene was used to enhance cat reporter gene expression in maize suspension cells without altering mRNA stability [173]. The first intron of the maize *Adh1* gene, positioned in its leader region, has been shown to amplify gene expression up to 100-fold compared to intronless constructs. This dramatic effect is attributed to the intron’s capacity to increase the accumulation and perhaps the quality of mRNA transcripts [170,174]. Similarly, studies on the maize *GapA1* gene highlighted the necessity of its native leader intron for any detectable transient expression in maize cells [175]. Additional examples include the leader intron of the maize *Shrunken-1* gene, which enhances reporter gene activity [176,177].

Some introns possess intrinsic promoter activity, enabling intron-mediated transcription initiation and expressing genes independently of conventional promoter sequences. This functional autonomy was demonstrated in monocots through the maize ubi1 intron, which actively directed GUS reporter expression in tritordeum inflorescences despite the absence of an exogenous promoter [178]. Interestingly, this effect seems to be cross-species working. Thus, leader introns from monocots (e.g., rice *actin1* intron) have been incorporated into maize transgenes and have exhibited significant enhancement of expression, underscoring the conserved nature of this regulatory mechanism in monocots [105].

### 3.6. Translation Enhancers

Located within UTRs, translation enhancers are sequence-specific *cis*-acting elements that upregulate gene expression by increasing translational efficiency. The broad ambiguity of the term “translation enhancer” makes it difficult to study them in plants, as there are various mechanisms of translation enhancement. These mechanisms include recruiting specific translation initiation factors, facilitating cap-independent translation, altering RNA secondary structure to enhance ribosomal scanning and access, or mimicking tRNA-like structures. Therefore, a thorough understanding of the mechanisms behind established “translation enhancers”, both from plant and viral origins, is essential for their effective use.

In eudicots, several viral 5′UTRs, like the Tobacco mosaic virus Ω leader, are known to dramatically boost protein yield when placed before a coding sequence [179,180]. A similar translation enhancement mechanism via the 5′UTR engineering with viral translation enhancers proves equally viable in monocots. Thus, the 73-nucleotide 5′UTR derived from the CaMV 35S promoter has been used in maize to boost luciferase activity by 35-fold [181]. Also, researchers can employ tandem viral enhancers (e.g., 2× or 4× CaMV 35S configurations) to amplify gene expression. These multiplexed enhancers remain functional in monocots, demonstrating significantly enhanced expression in rice and maize while conserving spatial expression profiles equivalent to the full CaMV 35S promoter in eudicots [182,183]. While the core promoter derived from CaMV 35S may be less optimal in monocots, the tandem enhancer repeats can still drive significant transcriptional activation, highlighting a critical modular difference between enhancers and core promoters. Another example of tandem enhancement is shown by the active ribosomal RNA complementary (ARC) element. A hexameric tandem repeat (6 × ARC) outperforms translation enhancers such as the Ω sequence, *OsADH*, and even 3 × ARC [184]. The region of 18S rRNA corresponding to ARC is conserved among rice, tobacco, maize, wheat, and human, suggesting its potential broad utility [185].

5′UTR enhancers are usually not cross-compatible through plant clades. So, 5′UTR sequences that enhance mRNA translation in eudicots may be less effective or completely inactive in monocots [136]. Matsui et al. evaluated in various plant species translational enhancers derived from plants and viruses, including the 5′UTRs from *N. tabacum*, *A. thaliana*, and *O. sativa* alcohol dehydrogenase genes (*NtADH*, *AtADH*, and *OsADH*, respectively), as well as the Ω sequence from Tobacco mosaic virus and the Tobacco etch virus leader (TEVL) [186]. They found that the *OsADH* 5′UTR functioned across all tested plant species, while the Ω and TEVL sequences were effective only in eudicots. Polysome profiling in rice led to the identification of several native 5′UTRs that act as strong translational enhancers, which can be used to improve transgene expression in monocots [136].

Cap-independent translation enhancers (CITEs) are another group of *cis*-acting regulatory elements that are known to enhance cap-independent translation initiation. We already mentioned IRES elements, which also provide cap-independent translation initiation. Unlike IRESs, which are exclusively 5′UTR-located, CITEs can be located both within the 5′UTR or 3′UTR. For 5′CITEs, the recognition of the 5′ cap by eIF4E still plays a major role in the mRNA recruitment. However, mRNA recruitment is still possible in the absence of this interaction [187]. The main difference between IRES and CITE is their mechanism of translation initiation. IRESs enable a form of cap-independent translation that employs direct internal ribosome recruitment, effectively bypassing the 5′ end and the need for ribosome scanning. In contrast, CITEs promote translation indirectly by recruiting translation initiation factors and facilitating mRNA circularization, thereby enhancing a scanning-dependent mechanism that still relies on recognition of the mRNA’s 5′ end [187]. The widespread distribution of CITE elements among plant viruses indicates their functional importance [188]. Although reports of cellular CITE elements in maize are currently lacking, a maize-specific virus harbors a 3′CITE element, demonstrating that this mechanism is functional in maize [189].

### 3.7. Start Codon Context Sequence

The start codon context sequence is a well-established *cis*-acting element that surrounds the translation initiation site. In eukaryotes, it is known as the Kozak sequence, which plays a pivotal role in ribosome recognition and regulation of translation initiation efficiency [150,190]. Although the Kozak sequence has certain patterns, common for all higher plants, eudicots and monocots have different preferences for nucleotide sequences surrounding the start codon [191]. This divergence likely results from differences in how translation initiation factors recognize and bind the mRNA template in these two major plant groups.

One of the common Kozak features is the preference for adenine residues from positions −21 to −1 relative to the start codon, with a peak of enhancement observed within the critical −5 to −1 window [157]. Particularly important for high translational efficiency is the presence of adenines at positions from −4 to −1 [191,192]. This preference was experimentally confirmed by the introduction of an optimal adenine-rich sequence to the start codon context, resulting in enhanced translational efficiency in both Arabidopsis and wheat cell extracts. In contrast to the positive role of adenine residues, thymine residues in this region consistently reduce translation efficiency [157]. Thorough research of all possible start codon context combinations in eudicot Arabidopsis revealed the best trinucleotides at position −3 in terms of codon context. So, the presence of AAG or GAG resulted in approximately 4-fold higher translation efficiency compared to the worst trinucleotide UGC [193]. In a similar study on monocot rice, the presence of the AGC sequence resulted in a 6-fold increase in translation compared to low-efficiency sequences such as UAU, UGA, or CGA. Also, ACC and UCC sequences demonstrated high translation efficiency [193].

Not only upstream of start codon nucleotides are important for translation efficiency determination, but the whole consensus sequence [194,195]. So, genomic analysis of plant coding sequences revealed the conserved guanine and cytosine residues at positions +4 and +5 relative to the start codon, respectively [196]. Thus, sequences (GC)/(AA)N**AUG**GC were identified as the consensus sequence for the translation initiation site signal in flowering plants [196].

The recent integration of deep learning and machine learning methods has provided a powerful framework for predictive modeling of translation initiation sites in maize [197]. These computational pipelines analyze diverse *cis*-regulatory features, including the Kozak sequence, and have aided in the rational design of improved translation initiation contexts. By combining these models with high-throughput translation assays, it becomes possible to predict and validate optimal sequence variants that may further enhance protein output in maize.

Taken together, the 5′UTR contains multiple regulatory elements that function in an interconnected manner. Interactions among these elements generate a multilayered regulatory framework that enables fine-tuned, context-dependent, and dynamic control of plant translational regulation, as summarized in Table 2.

## 4. Conclusions

The projected climate change is having an impact on crops as well. Plants have intrinsic mechanisms of gene expression adaptation, but the rate and severity of climate change may be too rapid and high for these adaptations to cope with. Thus, along with agricultural needs, adaptation to climate change requires the development of various transgenic plants. This requires a balance between robust trait expression and the preservation of wild-type agronomic performance. This requires precise regulation of recombinant protein production to prevent negative adaptation consequences and metabolic burden [107]. In such conditions, promoters remain the key point for genetic engineering. Therefore, a deeper understanding of monocot-specific promoter features, such as the Y patch motif, and the prevalence of multiple TATA boxes, as well as enhancer organization, will enable the rational design of tailored promoters for advanced synthetic biology applications in crop species.

Despite significant advances, transgenic research and applications in maize remain heavily dependent on a narrow group of promoters characterized over two decades ago [18]. Practical guidance for selecting promoters to maximize the predictability of expression levels in engineered maize lines is often elusive and can be oversimplified into general guidelines. Preference is typically given to promoters derived from the same taxonomic subgroup, provided they meet the researcher’s objectives, such as desired expression level, promoter length, ease of cloning, and other relevant factors. Although viral promoters can achieve high expression levels, their use in multiple-copy gene cassettes may lead to silencing, potentially compromising the intended expression. In cases where pleiotropic effects may occur, such as when expressing morphogenic factors to enhance regeneration, tissue-specific or inducible promoters are generally the preferred choice.

The development of synthetic promoters is a widely used approach for expanding the available promoter repertoire, as they allow for tunable gene expression and support the development of novel traits in plants [109,111,198,199]. Additionally, *cis*-regulatory engineering contributes to the design of synthetic promoters [200]. These efforts are increasingly powered by machine learning. For instance, the maize transcriptional regulatory network for 104 transcription factors was reconstructed using machine learning, revealing a scale-free, modular, and conserved architecture [201]. Furthermore, the integration of machine learning with massively parallel reporter assays is becoming increasingly common for *in silico* plant promoter prediction and the design of synthetic promoters with tunable strength [17,202,203,204]. Nevertheless, synthetic promoters require thorough *in vivo* testing to ensure functional consistency in the intended biological context.

5′UTR optimization is another important factor equivalent to promoter engineering for achieving the desired gene expression level in maize. 5′UTR modifications can achieve precise expression control unattainable through strong constitutive promoters alone [180]. Synthetic biologists can enhance the robustness, predictability, and yield of genetic circuits in maize by systematically incorporating key 5′UTR features, including an optimized start codon context, ribosomal scanning barriers (stem-loops/uORFs), and embedded *cis*-regulatory modules.

In addition to optimizing 5′UTRs, the design of new ones is actively developing, including computational design approaches [205,206]. The PlantRNA foundation model was designed to identify functional RNA motifs in plants, including both RNA sequences and structural motifs. It was successfully used to determine critical regions inside the 5′UTRs that significantly affect translation [207]. The application of such computational design algorithms to maize holds significant potential for generating customized 5′UTR sequences that maximize expression of target open reading frames. However, currently, empirically validated 5′UTRs remain the most reliable option for predictable transgene expression in maize. As an example, the intron-containing 5′UTR of ZmUbi1 promoter delivers robust constitutive expression, making it a benchmark for high-output transgenesis. In contrast, the relatively short 5′UTR from the CaMV 35S promoter is frequently utilized when a moderate expression level is required.

Finally, different combinations of 5′UTRs and promoters enable fine-tuning of expression levels, with up to a 10-fold expression range observed in maize cells when using the same promoter with various 5′UTRs [107]. Crucially, since expression level depends on the final construct design architecture, including promoter-5′UTR combination, each combination should be evaluated separately to verify its efficacy in a specific regulatory context. The necessity for such validation not only highlights the complex nature of transcriptional and translational regulation but also exposes fundamental gaps in our understanding of their precise regulatory interplay.

## Figures and Tables

**Table 2:** Plant 5′UTR regulatory elements with relevance to maize-specific gene regulation.

Biological/Biotechnological Applications	Representative Maize Examples	Plant-Specific Occurrence	Element
Supports mRNA stability and translation efficiency	Cap-0 form	Cap-0 form	5′ cap
Cap-independent translation under stress condition	*ZmHsp101* gene	Few confirmed examples in plant cellular mRNAs	IRES
Fine-tuning gene expression	Maize drought-responsive genes	Common	uORFs
Translational control during environmental stimuli; design of synthetic regulatory UTRs	Structured 5′UTRs in *ZmRab17* gene	Common	RNA secondary structure
Intron-mediated enhancement to increase transgene expression	Introns from *Adh1* and *Shrunken-1* genes	Common	Introns
Supports high-level gene expression	*ZmUbi1* 5′UTR	Identified in dicot and monocot plants	Translation enhancers
Optimization of translation initiation in gene constructs	Expected to follow the plant consensus sequence	Conserved among plants	Start codon context sequence

## Data Availability

No new data were created or analyzed in this study. Data sharing is not applicable to this article.
